# Supplementary material for: Resolving the full spectrum of human genome variation using Linked-Reads
Source: Genome Res. 2019 Apr;29(4):635–45. doi: 10.1101/gr.234443.118 (PMC6442396; doi:10.1101/gr.234443.118)
Supplement: Supplemental Material [file supp_gr.234443.118_Supplemental_longranger-2.2.zip › longranger-2.2.tar.bz2!/lr2.2-public/lib/go/src/code.google.com/vitess/docs/user-guide/client-libraries.html]

Vitess / Client Libraries


Toggle navigation

- Overview
- Guides
- Reference
- Blog
- GitHub

- #### Overview

  - What is Vitess
  - Scaling MySQL with Vitess
  - Key Concepts
- #### Getting Started

  - Run Vitess on Kubernetes
    - Custom Docker Build
  - Run Vitess Locally
- #### User Guide

  - Introduction- Client Libraries- Backing Up Data- Reparenting
        - Schema Management
        - VSchema Guide
        - Schema Swap (Tutorial)
        - Sharding
          - Horizontal Sharding (Tutorial, automated)
          - Horizontal Sharding (Tutorial, manual)
          - Sharding in Kubernetes (Tutorial, automated)
          - Sharding in Kubernetes (Tutorial, manual)
        - Vitess Sequences
        - MySQL Server Protocol
        - Vitess and Replication
        - Update Stream
        - Row Based Replication
        - Topology Service
        - Transport Security Model
        - Launching
          - Scalability Philosophy
          - Production Planning
          - Server Configuration
          - 2PC Guide
          - Troubleshooting
        - Upgrading
- #### Reference Guides

  - Vitess API- vtctl Commands
- #### Other Resources

  - Presentations- Blog- Roadmap
- #### Contributing

  - Contributing to Vitess- GitHub Workflow- Code Reviews
- GitHub

# Client Libraries

- #### Overview

  - What is Vitess
  - Scaling MySQL with Vitess
  - Key Concepts
- #### Getting Started

  - Run Vitess on Kubernetes
    - Custom Docker Build
  - Run Vitess Locally
- #### User Guide

  - Introduction- Client Libraries- Backing Up Data- Reparenting
        - Schema Management
        - VSchema Guide
        - Schema Swap (Tutorial)
        - Sharding
          - Horizontal Sharding (Tutorial, automated)
          - Horizontal Sharding (Tutorial, manual)
          - Sharding in Kubernetes (Tutorial, automated)
          - Sharding in Kubernetes (Tutorial, manual)
        - Vitess Sequences
        - MySQL Server Protocol
        - Vitess and Replication
        - Update Stream
        - Row Based Replication
        - Topology Service
        - Transport Security Model
        - Launching
          - Scalability Philosophy
          - Production Planning
          - Server Configuration
          - 2PC Guide
          - Troubleshooting
        - Upgrading
- #### Reference Guides

  - Vitess API- vtctl Commands
- #### Other Resources

  - Presentations- Blog- Roadmap
- #### Contributing

  - Contributing to Vitess- GitHub Workflow- Code Reviews

You can access your Vitess cluster using a variety of clients and
programming languages. Vitess client libraries help your client
application to more easily talk to your storage system to query data.

Vitess' service is exposed through a
proto3
service definition. Vitess supports gRPC,
and you can use the
proto compiler
to generate stubs that can call the API in any language that the
gRPC framework supports.

This document explains the client library strategy for Vitess.

## Core Principles

Vitess client libraries follow these core principles:

- Libraries fully implement the server API and provide access to all
  API methods, which support the following types of functions:
  - Transactions (begin, commit, rollback)
  - Queries targeted to a specific shards or groups of shards
  - Queries targeted based on sharding key values
  - Queries that monitor sharding configurations
  - MapReduce operations
- Libraries expose an identical set of well-documented error codes.
- Libraries provide a single-connection abstraction to the
  `vtgate` service. The connection object should be
  thread-safe, if applicable, and should support multiplexing for
  streaming queries, transactions, and other operations that rely
  on multiple queries.
- A low-level plug-in abstraction enables the transport to be plugged
  in so that the application can use different RPC frameworks. For
  instance, within Google, we use proto2 objects with an internal
  RPC system.  
  **Note:** If the proto3 library is available in
  your language, we recommend that your API calls use the data types
  generated from our .proto files.
- A high-level object abstraction allows the user to execute transactions
  without having to keep track of the database session.
- Libraries support `vtgateclienttest`, enabling you to
  fully unit-test all API calls. `vtgateclienttest` is
  a small server that simulates a real `vtgate` server
  and returns specific responses to allow for full client feature
  coverage.

## Language-specific considerations

- Each client library should support language-specific, idiomatic
  constructs to simplify application development in that language.
- Client libraries should integrate with the following language-specific
  database drivers. For example, current languages implement the following:
  - Go: database/sql package
  - Java: JDBC
  - PHP: PHP Data Objects (PDO)
  - Python: PEP 0249 DB API
- Libraries provide a thin wrapper around the proto3 service definitions.
  Those wrappers could be extended with adapters to higher level libraries
  like SQLAlchemy (Python) or JDBC (Java), with other object-based helper
  classes in the relevant language, or both.
- If a well-known MapReduce framework exists for the language, the client
  library should provide a data source and a data sink. For example, you
  can read data from Vitess inside a Hadoop MapReduce job and save the
  output into Vitess.

## Available libraries

### Go

The Go client interface is in the
"vtgateconn" package.

There are multiple implementations available. We recommend to use the
"grpc" implementation.
Load it by importing its package:

```
import "github.com/youtube/vitess/go/vt/vtgate/grpcvtgateconn"
```

When you connect to vtgate, use the
`DialProtocol` method
and specify "grpc" as protocol.
Alternatively, you can set the
command line flag "vtgate\_protocol"
to "grpc".

The Go client interface has multiple `Execute*()` methods for different use-cases
and sharding configurations. When you start off with an unsharded database, we
recommend to use the
ExecuteShards method
and pass "0" as only shard.

For an example how to use the Go client, see the end-to-end test
local\_cluster\_test.go.
From this test file, you can also reuse the "LaunchVitess" call to
instantiate a minimal Vitess setup (including a MySQL server). This way you can
test your application against an actual instance.

### Java

- Java client

### PHP

- PHP client

### Python

- Python client

Contact: vitess@googlegroups.com  **·**  
Announcements  **·**  
© 2017 Vitess powered by Google Inc
